# Decreased Fat Storage by *Lactobacillus Paracasei* Is Associated with Increased Levels of Angiopoietin-Like 4 Protein (ANGPTL4)

**DOI:** 10.1371/journal.pone.0013087

**Published:** 2010-09-30

**Authors:** Linda Aronsson, Ying Huang, Paolo Parini, Marion Korach-André, Janet Håkansson, Jan-Åke Gustafsson, Sven Pettersson, Velmurugesan Arulampalam, Joseph Rafter

**Affiliations:** 1 Department of Microbiology, Cell and Tumor Biology, Karolinska Institutet, Stockholm, Sweden; 2 Department of Biosciences and Nutrition (NOVUM), Karolinska Institutet, Huddinge, Sweden; 3 Department of Laboratory Medicine, Karolinska Institutet, Huddinge, Sweden; 4 Arla Foods Nordic Innovation, Stockholm, Sweden; 5 Department of Biology and Biochemistry, University of Houston, Houston, Texas, United States of America; New Mexico State University, United States of America

## Abstract

**Background:**

Intervention strategies for obesity are global issues that require immediate attention. One approach is to exploit the growing consensus that beneficial gut microbiota could be of use in intervention regimes. Our objective was to determine the mechanism by which the probiotic bacteria *Lactobacillus paracasei* ssp *paracasei* F19 (F19) could alter fat storage. Angiopoietin-like 4 (ANGPTL4) is a circulating lipoprotein lipase (LPL) inhibitor that controls triglyceride deposition into adipocytes and has been reported to be regulated by gut microbes.

**Methodology/Principal Findings:**

A diet intervention study of mice fed high-fat chow supplemented with F19 was carried out to study potential mechanistic effects on fat storage. Mice given F19 displayed significantly less body fat, as assessed by magnetic resonance imaging, and a changed lipoprotein profile. Given that previous studies on fat storage have identified ANGPTL4 as an effector, we also investigated circulating levels of ANGPTL4, which proved to be higher in the F19-treated group. This increase, together with total body fat and triglyceride levels told a story of inhibited LPL action through ANGPTL4 leading to decreased fat storage. Co-culture experiments of colonic cell lines and F19 were set up in order to monitor any ensuing alterations in ANGPTL4 expression by qPCR. We observed that potentially secreted factors from F19 can induce ANGPTL4 gene expression, acting in part through the peroxisome proliferator activated receptors alpha and gamma. To prove validity of *in vitro* findings, germ-free mice were monocolonized with F19. Here we again found changes in serum triglycerides as well as ANGPTL4 in response to F19.

**Conclusions/Significance:**

Our results provide an interesting mechanism whereby modifying ANGPTL4, a central player in fat storage regulation, through manipulating gut flora could be an important gateway upon which intervention trials of weight management can be based.

## Introduction

Despite continuing efforts to educate the public on the link between being excessively overweight and developing chronic diseases, the prevalence of obesity continues to increase (reviewed in [1]). This rapid increase and its significant health and economic burden have motivated a search for better prevention and treatment strategies. Alongside evaluation studies of popular weight loss regimens, scientific interest has also extended to secretory products such as adipokines, shown to influence aspects of pathogenesis of obesity-related diseases and weight loss. Here the physiology of fasting has become an issue since weight gain stems from an excess of caloric intake over expenditure. A critical event in the fasting response is its metabolic adaptations and the liberation of fatty acids from adipose tissue governed by numerous endocrine and cellular factors. One such factor is the Angiopoietin-like 4 protein (ANGPTL4, FIAF for fasting induced adipose factor, or PGAR for PPARγ angiopoietin related). ANGPTL4 is a circulating lipoprotein lipase (LPL) inhibitor and plays a key role in regulating deposition of triglycerides in adipocytes [2], [3]. ANGPTL4 is also a downstream target gene of peroxisome proliferator activated receptors (PPAR), the agonists of which are widely used for the treatment of type 2 diabetes and dyslipidemia [4], [5]. ANGPTL4 has been reported to be highly expressed in liver and adipose tissue [5], and plasma levels of the protein decrease on a chronic high-fat diet [4]. Interestingly, ANGPTL4 has also been shown to be susceptible to regulation by the gut microbiota. It was suggested that a conventional whole gut flora down regulated intestinal ANGPTL4, which promoted adiposity [6], [7]. It was also speculated that “Westernized” microbial ecology may function as a predisposing factor for obesity. This hypothesis is corroborated by findings of differences in recipient's body fat being dependent on the origin of a colonizing flora [8], and that diversity in the microbiota may contribute to subsequent fat storage [9].

In recent years, we have begun to understand the benefits of a well composed intestinal flora, emphasizing a role for health promoting pro- and prebiotics. Probiotics are living microbial food ingredients beneficial to health beyond basic nutrition. The most common and researched species belong to the genera *Lactobacillus*, *Bifidobacterium* and *Saccharomyces* [10]. Interestingly, there are some reports in the literature indicating possible anti-obesity effects of probiotic bacteria, although the underlying molecular mechanisms have not been revealed [11], [12], [13], [14]. In the current study, we examined the possibility that probiotic bacteria could target the fat storage regulator ANGPTL4 and, as a consequence, execute its modulatory effects. We have chosen to focus on *Lactobacillus paracasei* ssp *paracasei* F19 (F19) which is a gram-positive, non-spore forming bacterium initially isolated from human small intestine.

## Results

### F19 Supplementation to a High-fat Diet Decreases Stored Body Fat

**Figure 1:**
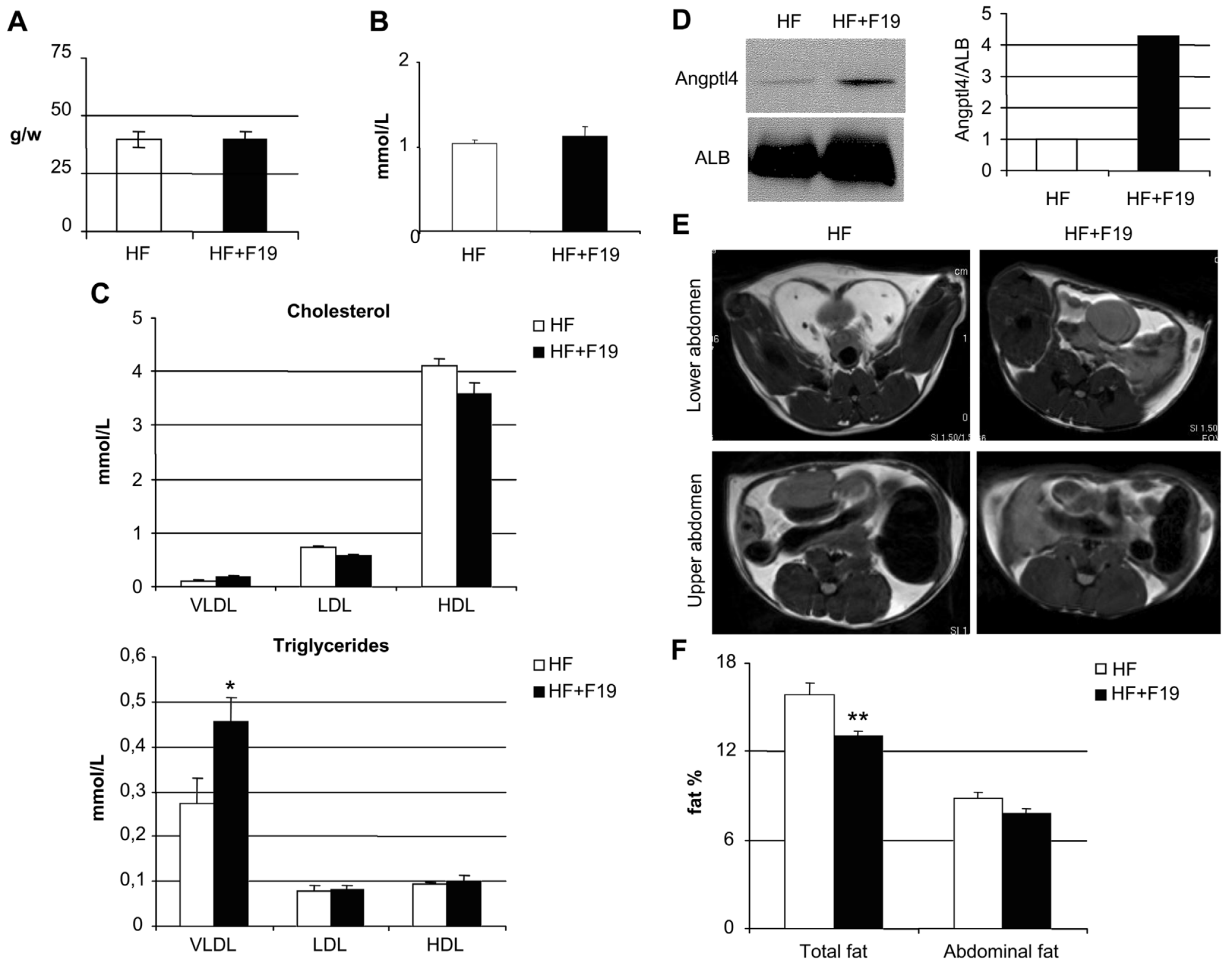
F19 supplementation decreases fat storage *in vivo*. A) The two groups (5 mice/group) of high-fat (HF) and high-fat supplemented with F19 (HF+F19) were pair-fed, referenced here by food consumption in grams/week. B) Free fatty acid content in the serum of the two groups. C) Lipoprotein profiles of both cholesterol and triglyceride contents of very low density lipoproteins (VLDL), low density lipoproteins (LDL), and high density lipoproteins (HDL). D) Western blot for full length ANGPTL4 in pooled serum (10 µg protein loaded) from HF and HF+F19 mice along with numerical representation of the same. E) Representative images from MRI visualizing fat depots (in white) in both the abdominal and visceral regions. F) Body fat percentages for the two groups. Stars represent P = 0.045 (*), P = 0.002 (**) using Student's t-test.

To investigate effects of F19 in a system of high fat diet (20%), we used normal SPF C57B/6J mice due to their propensity for weight gain. The two groups of mice were pair-fed to ensure that potential weight gain would only be affected by the dietary probiotic content (Figure 1A). After a 10 week diet regime, the serum of the two groups was analyzed for different lipid components. Free fatty acids were not affected by the presence of F19 (Figure 1B), while the triglyceride load of the lipoprotein VLDL (very low density lipoprotein) showed a modest but significant increase although cholesterol levels remained unchanged (Figure 1C). Circulating ANGPTL4 levels were upregulated in the F19 supplemented group (Figure 1D). Magnetic resonance imaging (MRI) showed a significantly reduced body fat profile with the presence of F19 (Figure 1E–F).

### Probiotic Bacteria Induce ANGPTL4 Expression in Colonic Cells

**Figure 2:**
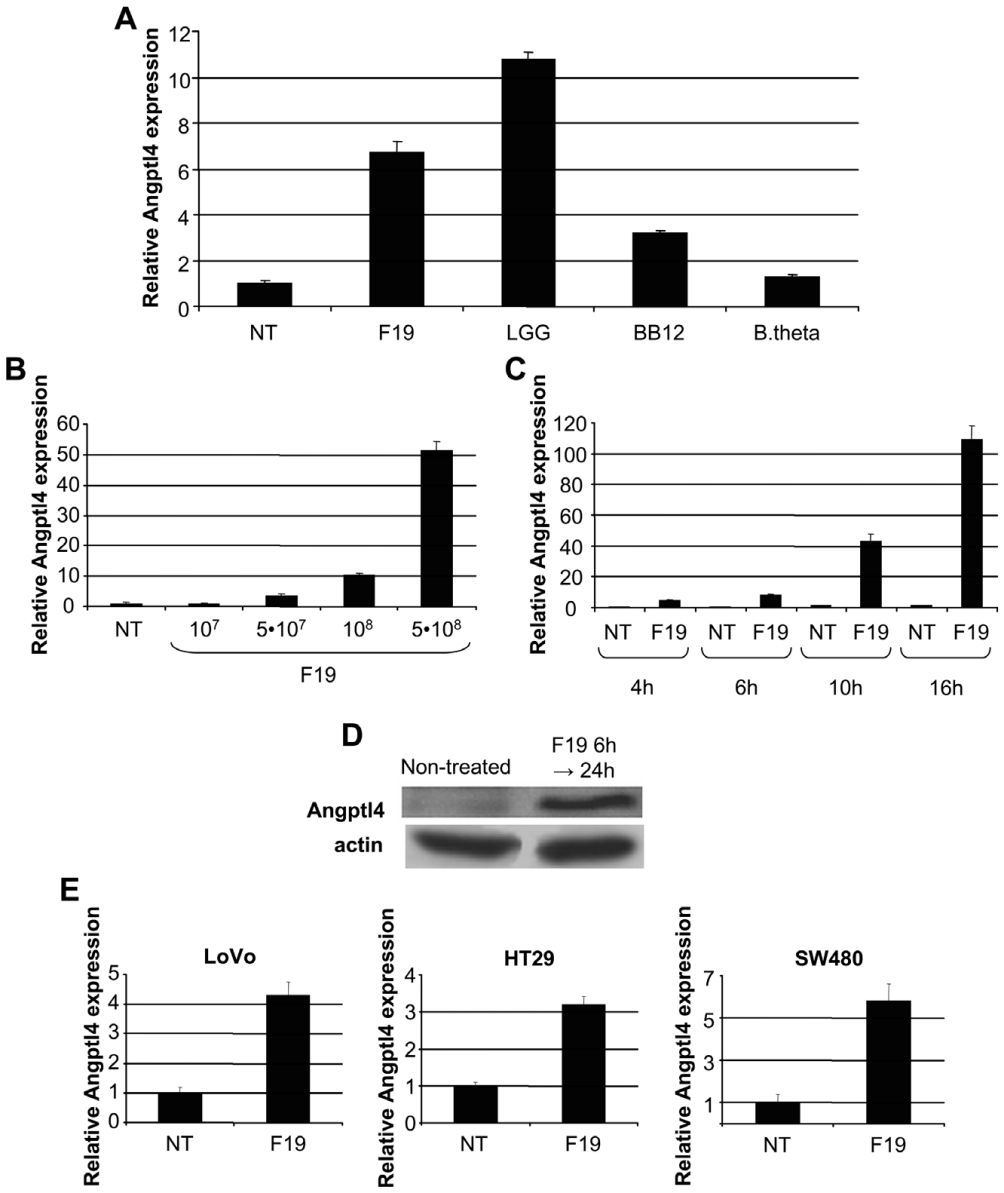
Probiotics upregulate ANGPTL4 expression in colonic cell lines. A) Real-time PCR of ANGPTL4 mRNA in HCT116 cells co-cultured with *Lactobacillus* F19 (F19; 10^7^/ml), *Bifidobacterium lactis* (BB12; 10^7^/ml) and *Bacteroides thetaiotaomicron* (B.theta; 10^7^/ml) respectively for 6 h were compared to non-treated (NT) control. B) Analysis of ANGPTL4 mRNA after 6 h stimulation with F19 at different concentrations in HCT116 cells. C) Time-course of F19 (10^8^) on ANGPTL4 mRNA expression in HCT116 cells. D) Western (50 µg) of full length ANGPTL4 in HCT116 cells treated with F19 for 6 h and collected after 24 h. Actin is shown as loading control. E) ANGPTL4 mRNA in the colon carcinoma cell lines LoVo, HT29 and SW480. Real-time PCR data are presented as means with standard errors. All data are representative of at least 3 independent experiments.

The colon carcinoma cell line HCT116 was used to determine effects of selected gut microbiota on ANGPTL4 expression in colonocytes. Cells were stimulated for 6h with the *Lactobacillus* F19, the *Bifidobacterium lactis* 12 (BB12), and the *Bacteroides thetaiotaomicron* (B.theta) (Figure 2A). Whereas F19 and BB12 generated an upregulation of ANGPTL4 mRNA, the commensal B.theta, was unable to stimulate expression in this cell line. The *Lactobacillus rhamnosus* GG (LGG) was also able to upregulate ANGPTL4 mRNA (data not shown). Further characterization of ANGPTL4 expression revealed that F19 upregulated ANGPTL4 mRNA in a dose and time dependent manner (Figure 2B–C). Elevated ANGPTL4 levels were further confirmed at the protein level in extracts from cells stimulated for 6 h with a subsequent 18 h accumulation period (Figure 2D). The issue of cell line exclusivity was addressed by quantifying ANGPTL4 mRNA after F19 stimulation in the colonic cell lines LoVo, HT29 and SW480, which all showed a similar upregulation of ANGPTL4 mRNA to that of HCT116 (Figure 2E).

**Figure 3:**
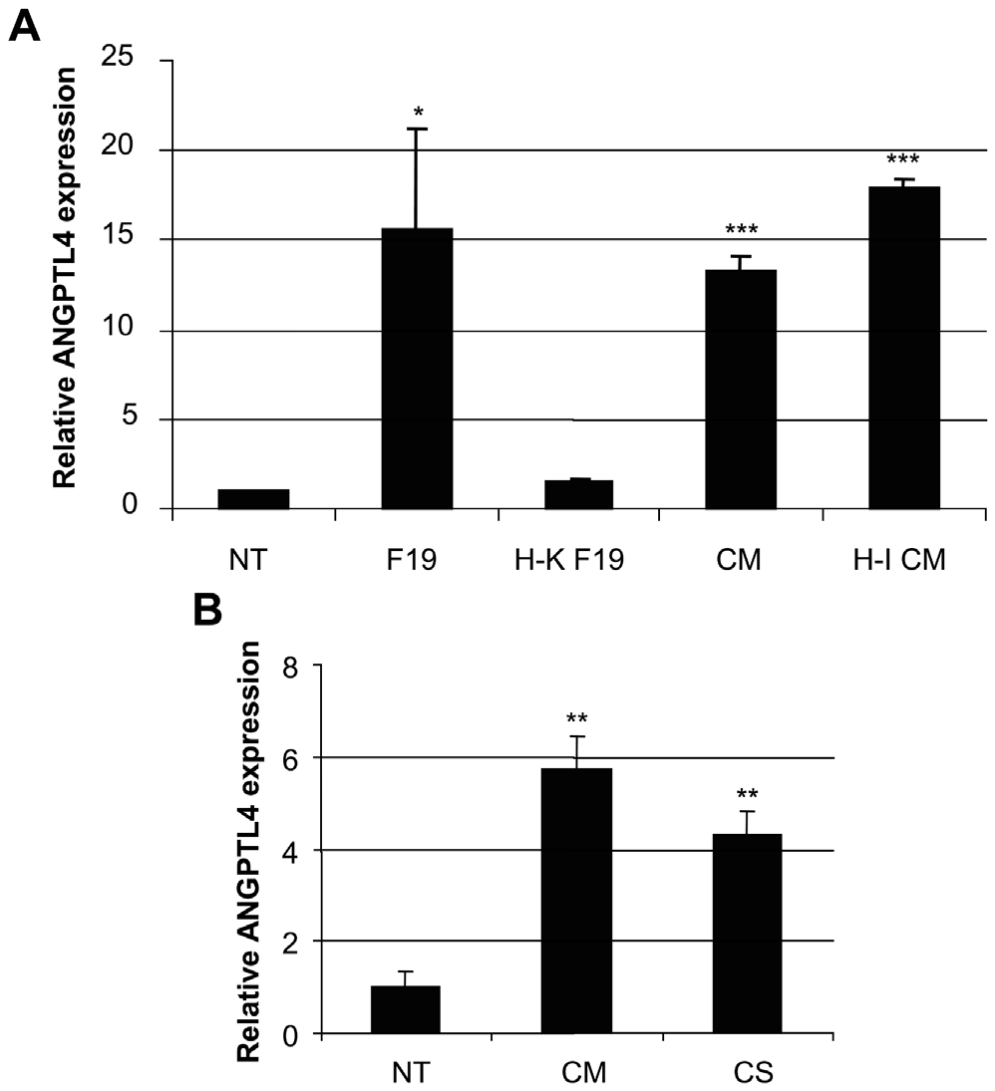
ANGPTL4 mRNA expression is regulated by F19 secreted factors. A) Real-time PCR of HCT116 cells stimulated for 6 h by live (F19) or heat-killed F19 (H-K F19) as well as fresh (CM) or heat-inactivated conditioned media of F19 (H-I CM) compared to non-treated (NT) control. B) Comparison between 6 h stimulation with conditioned media (CM) and F19 culture supernatant (CS) on ANGPTL4 expression. Bars signify means with standard errors. Results are representative of at least 3 independent experiments, and stars represent P<0.02 (*), P<0.01 (**), P<0.001 (***) (Student's t-test).

To elucidate the mechanism of ANGPTL4 gene induction, distinct components of the bacteria-cell interaction were studied. Heat-killed F19 could not mount an ANGPTL4 response, while conditioned media from bacteria interacting with cells, even when heat-inactivated, could (Figure 3A). The need for bacteria-cell contact for production of stimulatory molecules was also addressed. Here, we observed that supernatants of F19 grown without cells (CS) were as good as conditioned media (CM) where contact had occurred (Figure 3B).

### PPARα and PPARγ Signaling Pathways Play a Role in Induction of ANGPTL4 by F19

**Figure 4:**
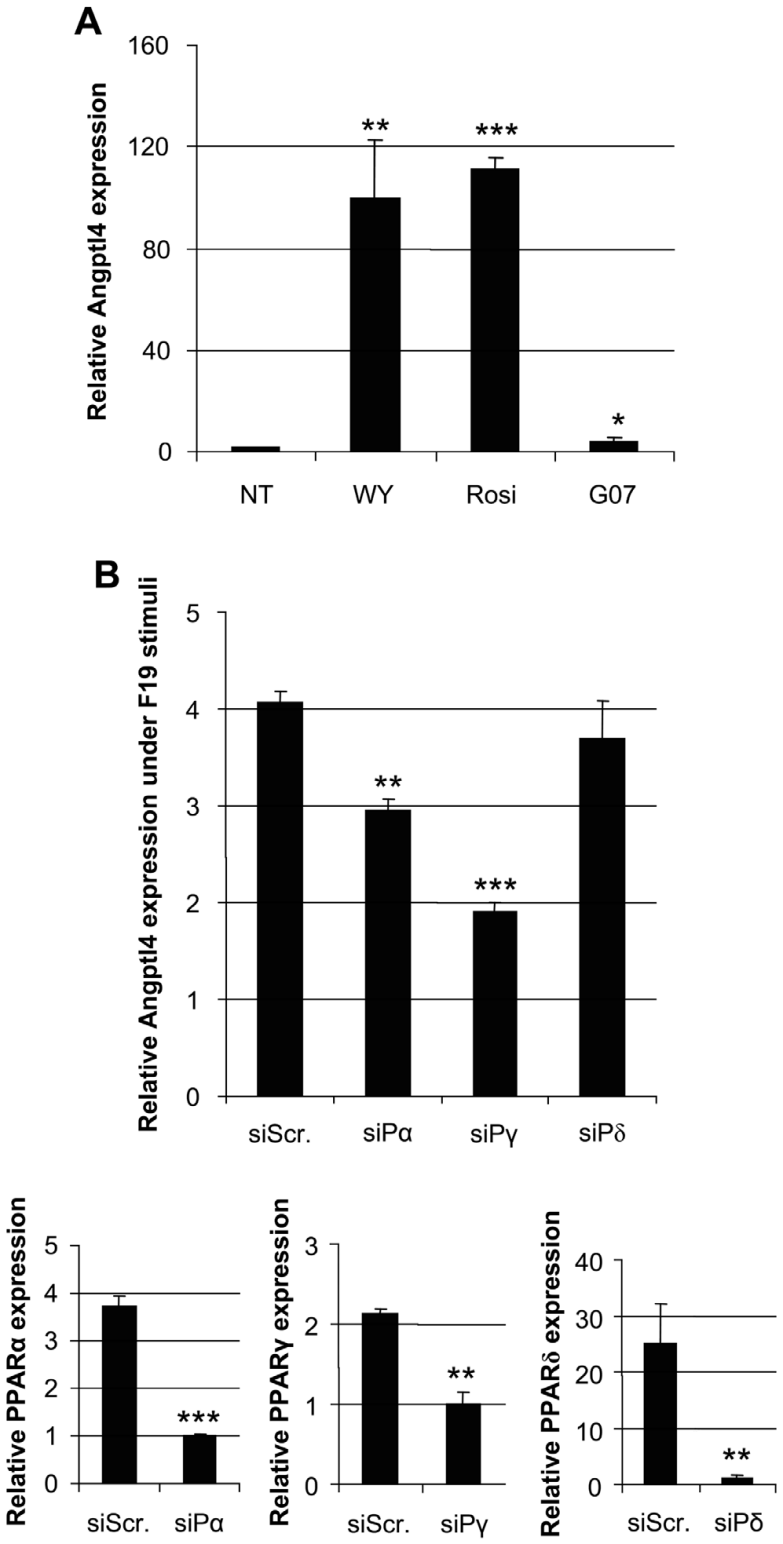
F19 mediated induction of ANGPTL4 gene expression may be mediated through PPARs. A) Stimulation of ANGPTL4 gene expression using the PPARα ligand WY-14643 (WY), PPARγ ligand Rosiglitazone (Rosi), and PPARδ ligand GW0742 (G07). B) Inhibition of ANGPTL4 response in HT29 cells after 6 h incubation with F19 by siRNA for PPARα, PPARγ, and PPARδ. Controls for siRNA efficiency of each inhibition are included as separate qPCR graphs. Expression data are presented with standard errors of the mean. Data are representative of at least 3 independent experiments, and stars represent P<0.05 (*), P<0.01 (**), P<0.001 (***) (Student's t-test).

PPAR specific ligand stimulation of HT29 cells resulted in increased amounts of ANGPTL4 transcripts, confirming it, with varying degrees, as a target for all three nuclear receptors (Figure 4A). When PPARγ and PPARα were downregulated by siRNA in HT29 cells induction of ANGPTL4 expression by F19 was markedly reduced (Figure 4B), revealing a partial nuclear receptor dependency for this gut microbiota mediated effect. Downregulating PPARδ did not influence the ability of F19 to upregulate ANGPTL4 (Figure 4B). Combinatorial transfection of the different siRNAs did not result in cooperative effects on ANGPTL4 expression (data not shown).

### Mono-colonization of Germ-free Mice with F19 Elevates ANGPTL4 Protein in Serum

**Figure 5:**
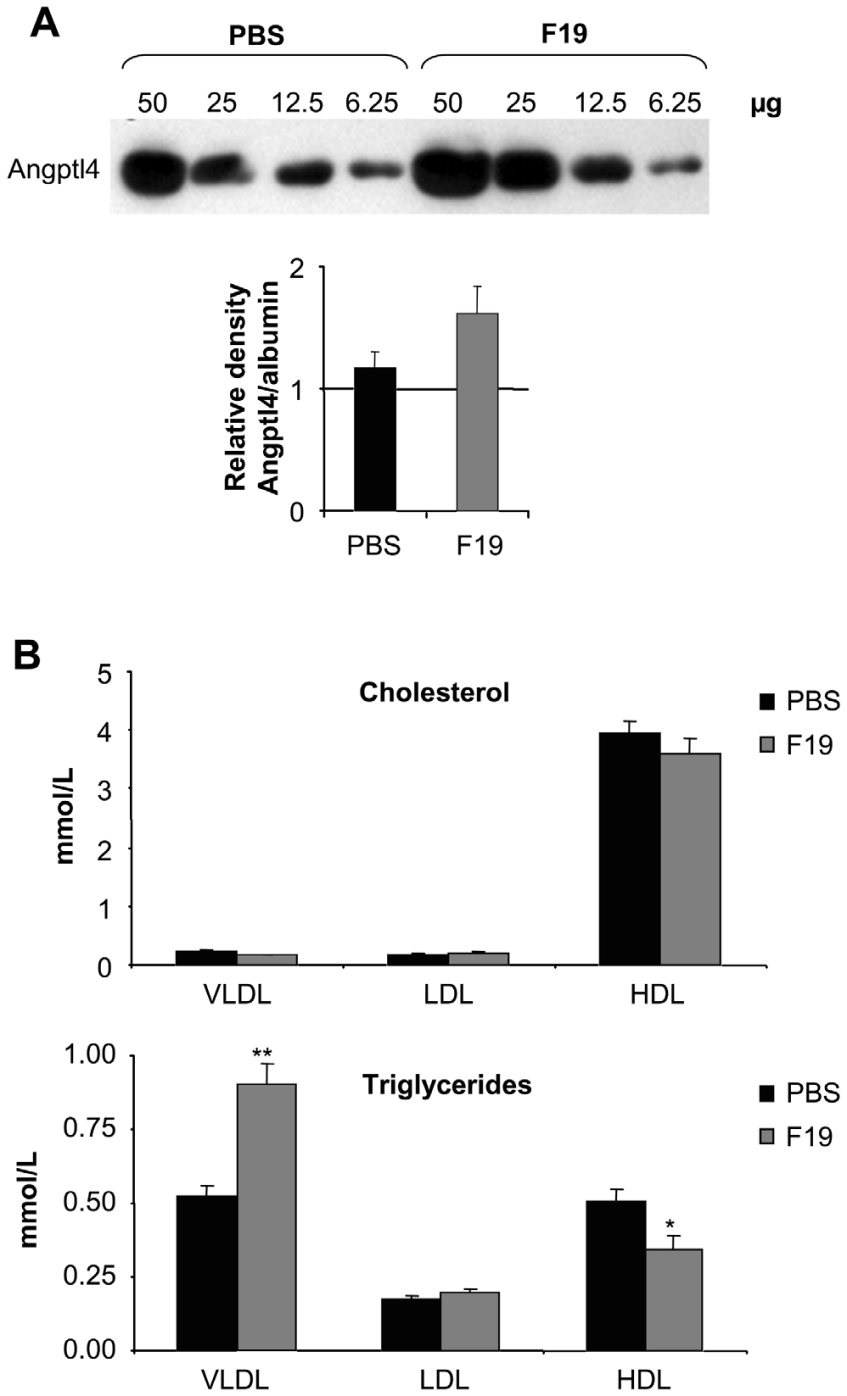
F19 monocolonization in germ-free mice increases ANGPTL4 protein levels in serum. A) Western blot for full length ANGPTL4 levels in a serially diluted (50, 25, 12.5, 6.25 µg protein) serum pool of control (PBS) and mono-infected mice (F19), along with a collated numerical representation of the same western corrected for loading. B) Cholesterol and triglyceride profiles of very low density lipoprotein (VLDL), low density lipoprotein (LDL), and high density lipoprotein (HDL) in control (PBS) and mono-infected serum (F19). Indicated bars represent the average value of each data set, n = 6, while stars represent P = 0.045 (*), P = 0.002 (**) (Student's t-test).

To confirm a possible regulatory effect *in vivo*, we chose germ-free (GF) NMRI mice on normal chow and exposed them to F19. No adverse side effects on general health were observed during the 2 week colonization period, after which serum was collected from the sacrificed mice for further investigation. Serum levels of ANGPTL4 protein revealed an increasing trend within 2 weeks of colonization with F19 (Figure 5A). Interestingly and in consonance with this, an increase in triglycerides in the VLDL fraction was seen, as possible consequence of LPL inhibition, whereas the mono-colonization with F19 had no effect on cholesterol content in any of the lipoprotein fractions (Figure 5B).

## Discussion

In a key paper, it was reported that the gut microbiota in obese humans and mice differ from those in lean individuals [9]. Here the notion was raised, for the first time, that these bacteria are involved in body weight regulation and may be a factor in the obesity epidemic. This high-lighted the intriguing question as to whether we could consider a “rational food design” approach, targeting the gut microbiota, as opposed to a drug-based approach to address the problem of overweight. Such an approach, in combination with altered food intake, has an advantage in reaching a larger number of individuals in a cost effective manner. The use of dietary components, together with selected normal commensal bacteria, to regulate fat metabolism is one approach where microbial entities are used to affect complex downstream homeostatic presets.

A major goal of the present study was to address underlying mechanisms for the reported effects of probiotics on adiposity. To this end, we now confirm a clear difference in stored adiposity between F19 and control mice on a high fat diet, and furthermore observe an elevation of serum triglycerides possibly as a consequence of the increase in circulating ANGPTL4. Indeed, similar effects on lipoproteins have been seen in ANGPTL4 transgenic mice, strengthening the probability that increased triglycerides is a direct function of increased levels of circulating ANGPTL4 [15]. Free fatty acids were not elevated indicating that the mice have not developed any symptoms of metabolic syndrome in response to either the diet or the elevated triglyceride levels. Furthermore, feeding of F19 did not alter the levels of the lipoprotein fractions and thus did not result in a more atherogenic lipid profile, since isolated elevation of triglycerides is not in itself atherogenic in rodents [16], [17]. In extension, it would be interesting to investigate F19 action in a truly obese model system to elucidate how fat storage might be affected under more extreme conditions.

We show that ANGPTL4 expression can be induced by an array of probiotic strains including F19. In contrast, the anaerobic commensal *B. thetaiotaomicron*, is unable to induce ANGPTL4 which is in line with the reported inability of whole flora to increase ANGPTL4 expression [6]. At present, the *Lactobacillus* F19 seems to be a better inducer of the LPL inhibitor than the *Bifidobacterium* BB12. Further experiments on strain differences are highly warranted.

In order to address the mechanism of action of F19, we monitored ANGPTL4 expression as a result of bacterial presence. Inability of heat-killed F19 to generate a response and the redundancy of bacteria-cell interaction, allowed us, based on these experiments, to currently exclude bacterial wall components as the sole source of stimuli. Conditioned media from F19 co-cultured with HCT116 cells was, on the contrary, sufficient, even when heat-inactivated, to increase ANGPTL4 expression. Taken together, these data suggest that the *Lactobacillus* F19 secreted, as yet uncharacterized, heat stable molecules could be responsible for the observed effects on ANGPTL4.

ANGPTL4 is a target gene of the PPARs [18] which was confirmed by specific ligand stimulation. When PPARα and PPARγ were abrogated by siRNA, induction of ANGPTL4 by F19 was considerably compromised. This implies that one route by which F19 signals for ANGPTL4 increase is via the PPARs, which have crucial roles in energy homeostasis and adipogenesis (reviewed in [19]). Interestingly, we have previously shown that expression and function of nuclear receptors such as the PPARs, can be regulated by gut microflora regarding both expression and function [20], [21]. However, despite ablation of PPARα and PPARγ, we still observed ANGPTL4 expression indicating that additional players are also regulating ANGPTL4 expression.

Finally, we were able to confirm our results in another animal model. Monocolonizing GF NMRI mice with F19 for two weeks resulted in increased circulating ANGPTL4 protein compared to non-infected GF mice. In addition, the signature increase in triglycerides was also seen, an observation compatible with an LPL inhibition caused by the increase in ANGPTL4 serum levels. It should be noted that the altered serum lipoprotein profile observed in the F19 treated mice was not of such a magnitude that might lead to any long-term detrimental effects.

While mindful of some of the limitations of this study, we believe that our findings provide a fundamental rationale for further studies using select probiotic microbes in an overweight setting. We are just beginning to comprehend how variables such as food intake, exercise, hormonal control, and genetic variation interact and result in a given phenotype. Our results open the intriguing possibility that modifying LPL activity through ANGPTL4, central to fat storage regulation, by manipulating the gut flora could be one important mechanism by which various interventions may modulate body fat storage.

## Materials and Methods

### Mice, Cell Lines and Reagents

Ten ten-twelve week old SPF (specific pathogen free) C57B/6J male mice, divided into two groups, were put on a 10 week diet of modified R36 Lactamin chow containing 0.2% cholesterol, 20% cocoa fat, +/− F19 (2×10^9^ cfu/g feed), while kept in 12 h light cycles. The two groups were pair-fed for the duration of the diet, and sacrificed after whole body magnetic resonance imaging was carried out.

Twelve ten to twelve week old germ-free (GF) NMRI mice divided into two groups were maintained on autoclaved R36 Lactamin chow (Lactamin, Sweden) while kept in 12 h light cycles. *Lactobacillus* F19 was cultured on MRS plates and used to colonize GF NMRI mice for a period of two weeks in a concentration of 10^9^ per animal by one-time gavage. The colonized mice were sacrificed by cervical dislocation along with age matched GF PBS gavaged controls. From each animal the cecal content was cultured as treatment control, which confirmed positive colonization of F19 gavage as well as continued germ-free conditions after control PBS ingestion. Animal husbandry was in accordance with institutional guidelines at Karolinska Institutet and all animal experiments were approved by the ethical committee in Stockholm, Sweden (Stockholms norra djurförsöksetiska nämnd, N 107/07).

The human colorectal adenocarcinoma cell lines HCT116 (CCL-247, ATCC), LoVo (CCL-229, ATCC), HT29 (HTB-38, ATCC) and SW480 (CCL-228, ATCC) were grown and maintained according to supplier's recommendations.

PPARα ligand WY-14643 (used at 100 µM), PPARγ ligand Rosiglitazone (used at 5 µM), and PPARδ ligand GW0742 (used at 1 µM) were all products from Cayman.

### Bacteria and Co-Culture

The bacterial strains *Bifidobacterium lactis 12* and *Lactobacillus paracasei* subsp. *paracasei* F19 were obtained from Arla Foods AB (Stockholm, Sweden) while *Bacteroides thetaiotaomicron* was a lab stock (CFGR, Karolinska Institute).

F19 was always pre-cultured for 6–8 h at 37°C on a rotating platform (225 rpm). Pre-cultures were then added to pre-warmed deMan Rogosa Sharpe (MRS) medium (dilution 1∶20). The cultures were cultivated overnight prior to use. Bacterial concentration at OD_600nm_ was determined: 1 OD_600nm_ = 1×10^8^ F19/ml. The required amount of F19 was resuspended in an appropriate volume of the respective pre-warmed medium without antibiotics.

Co-culture was prepared by washing the colonic cells with warm PBS. Cells were incubated with 2×10^7^/ml of F19 or with medium alone. The experiment was terminated by thoroughly washing the plates with ice-cold PBS.

To determine the actual bacterial concentration the medium was diluted 1∶10^7^, 1∶10^8^, 1∶10^9^ in MRS medium for F19. Suspension was put on MRS plate and incubated at 37°C overnight. The initial bacterial concentration was calculated from the number of colonies.

As a control, F19 was heat-inactivated by incubating at 80°C for 30 minutes. The same amount of heat inactivated bacteria as used in the co-culture with live bacteria was suspended in 5 ml of respective medium and added to the washed cells. Conditioned medium was prepared by incubating F19 and HCT116 together, after 6 hours medium was collected and filtered (pore size: 0.2 um), whereas culture supernatant was collected from F19 in media without presence of cells. Conditioned medium was heat inactivated by boiling (100°C, 10 minutes).

### Real-Time PCR

RNA was prepared using the Qiagen RNeasy Mini Kit following the manufacturer's protocol and cDNA was synthesised using the cDNA synthesis kit from Invitrogen according to protocol. Semi-quantitative, SYBR Green based (Applied Biosystems) real-time PCR was used to detect transcripts. Forward and reverse primers were mixed at equal concentration and used at a final concentration of 0.2 µM. Beta-actin Fw: 5′-CCTGGCACCCAGCACAAT-3′, Rv: 5′-GCCGATCCACACGGAGTACT-3′; ANGPTL4 Fw: 5′- AAAGAGGCTGCCCGAGAT -3′, Rv: 5′-TCTCCCCAACCTGGAACA-3′. Each experiment was carried out in sample duplicates. The mRNA levels of each sample were determined in triplicates. Real-time PCR was performed using the ABI 7500 System for data acquisition and analysis by using the ABI 7500 System Sequence Detection software. Data is presented as mean values with standard errors.

### Western Blot

Cells were treated according to figure specifications and lyzed in Schindler lysis buffer (50 mM Tris pH 8; 0.1 mM EDTA; 0.5% NP-40; 10% Glycerol; 150 mM NaCl; 10 nM okadaic acid; 5 mM sodium fluoride; 400 µM sodium vanadate; 1x Complete (Roche, Germany); 1 mM phenylmethanesulphonylfluoride). The ANGPTL4 antibody (409800; Zymed; California, USA), raised against an internal region of the protein, was used to detect full length protein at a 1∶500 dilution, Actin (sc-1616) was used at 1∶1000, and Albumin (sc-50536) at 1∶2000, while immunodetection was carried out by an appropriate secondary peroxidase-conjugated antibody (DAKO A/S, Denmark) followed by chemiluminescence (ECL, Amersham, UK).

### SiRNA

Elimination of PPARα, PPARγ, and PPARδ transcripts from HT29 cells was accomplished by Dharmacons readymade siRNA products (SMARTpool Human PPARG/A/D). Transfection was carried out according to manufacturer's protocol using the DharmaFECT 4 reagent with a final siRNA concentration of 0.5 µM/cm^2^.

### Lipoprotein Analysis

Total cholesterol and triglyceride content in lipoprotein fractions were determined by size exclusion chromatography on 2.5 µl of individual plasma samples using a Superose 6 PC 3.2/30 column (GE Healthcare Bio-Sciences AB, Uppsala, Sweden) as described [22]. The respective lipoprotein fraction's lipid concentrations were calculated after integration of the individual chromatograms.

### Magnetic Resonance Imaging and Analysis

*In vivo* magnetic resonance imaging (MRI) measurements were made under 1.5–2% isoflurane in O_2_ anesthesia. Fat distribution was measured in every mouse after the 10th week of the diet period. All *in vivo* MRI were performed on a Bruker 4.7-T field strength magnet and a 40-cm horizontal bore diameter (Bruker Biospec Avance 47/40; Bruker, Karlsruhe, Germany) equipped with a commercially available circular resonator (Bruker) with an inner diameter of 24 mm for RF pulse application and signal detection. Body temperature was maintained using a temperature-controlled air stream around the body of the mouse. For measurement of whole body adiposity, the main sequence employed was a Bruker implementation of rapid acquisition with relaxation enhancement (RARE) imaging [23]. Briefly, the mouse was laid on a support and positioned in the center of the coil. A total of ∼ 40 contiguous 1.5-mm-thick transversal slices covering the mouse body were recorded. Image analysis was carried out with Paravision 3.01 image analysis software (Bruker). As a result, regional changes in fat were assessed on the basis of total, intra-abdominal, or visceral and subcutaneous fat depots. The abdominal fat is the sum of visceral and subcutaneous fat within the abdominal region. MRI-visible visceral fat comprises omental, retroperitoneal, and mesenteric fat depots. Two-dimensional image series were then imported into Biomap platform (Boulder, CO) for pixel counting-based determination of fat volumes. A signal threshold was used after a Gauss filter a maximum likelihood, and a class-select interaction was applied to exclude all nonfat tissues in each slice. A density factor of 0.9 g/ml was used to convert fat volumes (in ml) into fat mass (in g).
